# Elimination of Tumorigenic Human Pluripotent Stem Cells by a Recombinant Lectin-Toxin Fusion Protein

**DOI:** 10.1016/j.stemcr.2015.02.016

**Published:** 2015-04-09

**Authors:** Hiroaki Tateno, Yasuko Onuma, Yuzuru Ito, Fumi Minoshima, Sayoko Saito, Madoka Shimizu, Yasuhiko Aiki, Makoto Asashima, Jun Hirabayashi

**Affiliations:** 1 Research Center for Stem Cell Engineering, National Institute of Advanced Industrial Science and Technology, Tsukuba Central 2, 1-1-1 Umezono, Tsukuba, Ibaraki 305-8568, Japan; 2 Research Center for Stem Cell Engineering, National Institute of Advanced Industrial Science and Technology, Tsukuba Central 4, 1-1-1 Higashi, Tsukuba, Ibaraki 305-8562, Japan

## Abstract

The application of stem-cell-based therapies in regenerative medicine is hindered by the tumorigenic potential of residual human pluripotent stem cells. Previously, we identified a human pluripotent stem-cell-specific lectin probe, called rBC2LCN, by comprehensive glycome analysis using high-density lectin microarrays. Here we developed a recombinant lectin-toxin fusion protein of rBC2LCN with a catalytic domain of *Pseudomonas aeruginosa* exotoxin A, termed rBC2LCN-PE23, which could be expressed as a soluble form from the cytoplasm of *Escherichia coli* and purified to homogeneity by one-step affinity chromatography. rBC2LCN-PE23 bound to human pluripotent stem cells, followed by its internalization, allowing intracellular delivery of a cargo of cytotoxic protein. The addition of rBC2LCN-PE23 to the culture medium was sufficient to completely eliminate human pluripotent stem cells. Thus, rBC2LCN-PE23 has the potential to contribute to the safety of stem-cell-based therapies.

## Highlights

• rBC2LCN-PE23 binds to and is internalized by human pluripotent stem cells
• rBC2LCN-PE23 eliminates human pluripotent stem cells, but not differentiated cells
• rBC2LCN-PE23 should contribute to the safety of stem-cell-based therapies

## Graphical Abstract

**Figure:**
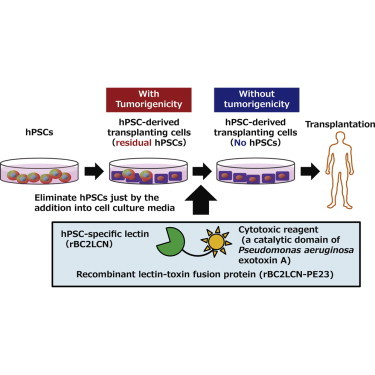

## Teaser

The application of human pluripotent stem cells in regenerative medicine is hindered by the tumorigenic risk from residual human pluripotent stem cells. In this article, Tateno and colleagues present a method to eliminate tumorigenic human pluripotent stem cells from a mixed cell population using a recombinant lectin-toxin fusion protein, termed rBC2LCN-PE23.

## Introduction

Human pluripotent stem cells (hPSCs), such as human embryonic stem cells (hESCs) (Thomson et al., 1998) and human induced pluripotent stem cells (hiPSCs) (Takahashi et al., 2007), offer immense potential as cell sources for cell-based therapies because of their capacity for unlimited self-renewal and pluripotent differentiation. In particular, hiPSCs are creating great expectations not only for regenerative medicine, but also for disease modeling and drug development, as they can be generated from various adult somatic cells simply by introducing reprogramming factors. Enormous efforts have been undertaken to establish hPSC-based therapies for a variety of degenerative diseases (Garber, 2013). Recently, the first in-human clinical trial using hiPSC-derived retinal pigment epithelium was conducted by RIKEN Center for Developmental Biology in Kobe to treat the wet form of age-related macular degeneration (Kamao et al., 2014). However, although the clinical and industrial application of hPSC-based cell therapy is becoming an increasingly realistic prospect, a major safety concern still exists, as residual hPSCs in differentiated cell populations could form tumors in recipients (Ben-David and Benvenisty, 2011; Goldring et al., 2011; Lee et al., 2013a).

Over the past several years, the tumorigenicity risks of hPSCs have been highlighted in a number of animal studies (Hentze et al., 2009; Kawai et al., 2010; Lee et al., 2009; Roy et al., 2006; Yamashita et al., 2011). As few as 100 hPSCs have been reported to be sufficient to produce a teratoma (Gropp et al., 2012; Hentze et al., 2009). Therefore, complete elimination of hPSCs from the final cell products without compromising their viability, safety, efficacy, and functional properties is a prerequisite for clinical application of hPSC-based therapy. It is also important to remove residual hPSCs from hPSC-derived cells to establish disease models. Several strategies to remove residual hPSCs from differentiated cell cultures have been reported, including the introduction of suicide genes into hPSCs (Schuldiner et al., 2003), selective killing using cytotoxic antibodies (Ben-David et al., 2013b; Choo et al., 2008; Tan et al., 2009) and chemical inhibitors (Ben-David et al., 2013a; Lee et al., 2013b; Richards et al., 2014; Vazquez-Martin et al., 2012), cell sorting using hPSC-specific antibodies (Ben-David et al., 2013b; Tang et al., 2011) and lectins (Wang et al., 2011), and glucose deprivation in the cell culture medium (Tohyama et al., 2013). However, all of these methods have some limitations in terms of specificity, throughput, efficacy, and safety. The development of alternative strategies based on different mechanisms therefore is warranted.

Previously, we performed comprehensive glycome analysis of a large number of hPSCs (114 types of hiPSCs and nine types of hESCs) using high-density lectin microarrays. We found that a lectin designated rBC2LCN (recombinant N-terminal domain of BC2L-C lectin derived from *Burkholderia cenocepacia*) binds to various types of hiPSCs and hESCs, but not to differentiated somatic cells (Tateno et al., 2011). rBC2LCN is a useful hPSC probe, which strongly stains formaldehyde-fixed hPSCs at the cell membrane (Onuma et al., 2013). In addition, it allows live staining of hPSCs after supplementation of the culture medium, with no visible signs of toxicity. The staining is specific to undifferentiated cells, and rapidly diminishes upon their differentiation. Detailed specificity analysis revealed that rBC2LCN binds to the defined glycan structure, Fucα1-2Galβ1-3GlcNAc/GalNAc (Sulák et al., 2010; Tateno et al., 2011). Among the *N*- and *O*-glycans isolated from 201B7 hiPSCs, a core2-type *O*-glycan, Fucα1-2Galβ1-3(Galβ1-4GlcNAcβ1-6)GalNAc, was found to contain the above glycan structure (Hasehira et al., 2012). rBC2LCN exhibited a significant affinity (*K*_a_ of 2.5 × 10^4^ M^−1^) to this core2-type *O*-glycan, demonstrating that it is a glycan ligand of rBC2LCN (Tateno et al., 2013). Furthermore, podocalyxin, a type1 transmembrane protein, was identified as a predominant glycoprotein ligand of rBC2LCN (Tateno et al., 2013). Podocalyxin is a hyperglycosylated sialomucin containing a mucin domain with >100 putative *O*-glycosylation sites, five potential *N*-linked glycosylation sites, and three putative glycosaminoglycan sites (Kershaw et al., 1997). Due to its heavily glycosylated nature, the apparent molecular weight of podocalyxin expressed on hPSCs is >200 kDa, despite the calculated molecular weight of 55 kDa (Tateno et al., 2013). Although the binding affinity of rBC2LCN to the core2-type *O*-glycan is relatively low, that to 201B7 hiPSCs is as high as antibody (*K*_a_ = 5 × 10^8^ M^−1^), presumably due to the high-density display of the glycan ligand on hPSCs, the so-called “glycoside cluster effect” (Dam and Brewer, 2010; Dam et al., 2009). Most recently, the hyperglycosylated podocalyxin was found to be secreted into cell culture media. Taking advantage of this phenomenon, a noninvasive and quantitative detection system of tumorigenic hPSCs in transplanting cells using cell culture media was developed (Tateno et al., 2014).

Based on these principles, we speculated that the use of rBC2LCN as a cargo of cytotoxic agent would provide an efficient strategy to eliminate hPSCs. Here we generated a recombinant lectin-toxin fusion protein by fusing rBC2LCN with 23 kDa of a catalytic domain of *Pseudomonas aeruginosa* exotoxin A (rBC2LCN-PE23) for the targeted elimination of hPSCs. rBC2LCN-PE23 could be produced as a soluble form in *E. coli* at ∼10 mg/l culture and purified to homogeneity using one-step affinity chromatography. It showed similar glycan binding specificity to rBC2LCN, and, when added to culture medium, bound to hiPSCs and was internalized by the cells. hiPSCs as well as hESCs were eliminated after 24 hr culture in the presence of rBC2LCN-PE23, although no effect was observed for retinoic acid (RA)-treated hiPSCs, human dermal fibroblasts (hFibs), and human adipose-derived mesenchymal stem cells (hADSCs). Thus, rBC2LCN-PE23 could be used as a reagent to remove tumorigenic hPSCs from differentiated cell populations, lowering the risk of teratoma formation by its installation into hPSC-based regenerative medicine.

## Results

### Production of rBC2LCN-PE23

**Figure 1:**
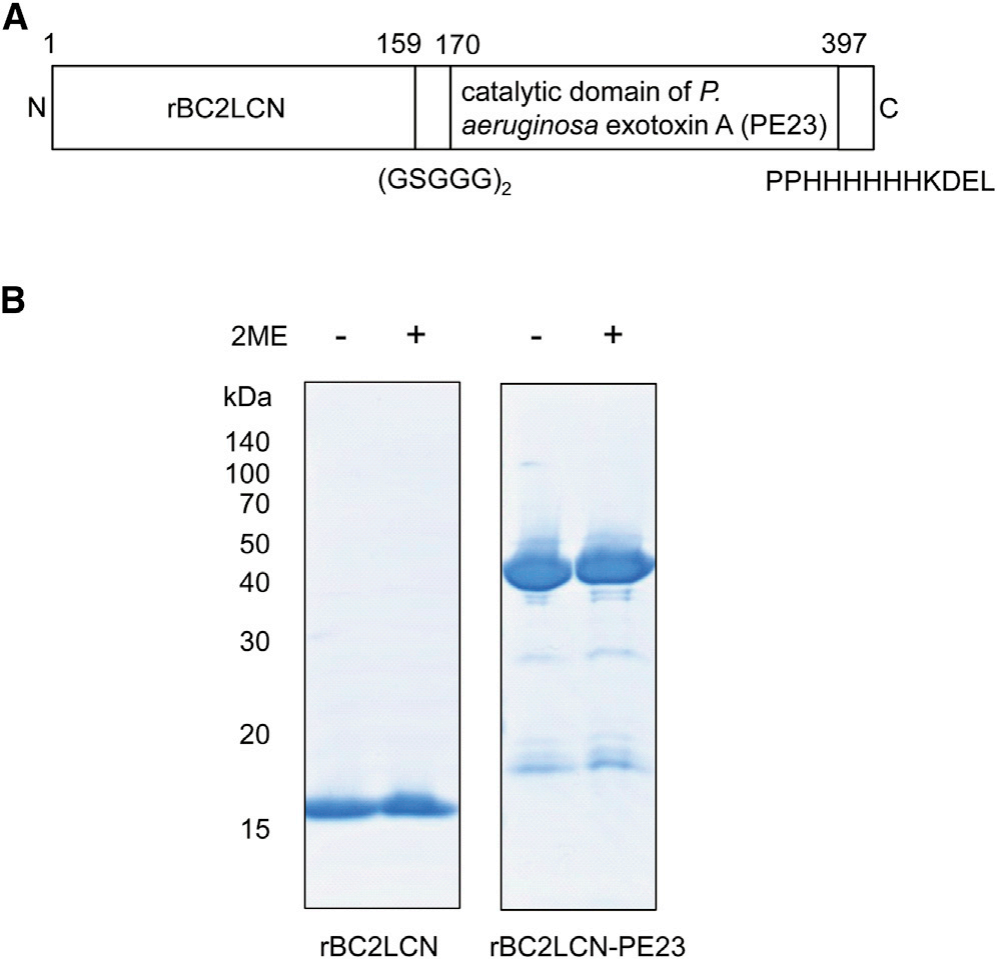
Production of rBC2LCN-PE23 (A) Domain structure of rBC2LCN-PE23. rBC2LCN-PE23 consists of an *N*-terminal rBC2LCN (156 amino acid residues), ten amino acid linker (GSG_3_)_2_, and a catalytic domain of *Pseudomonas aeruginosa* exotoxin A (399–613 amino acid, 215 amino acid residues) carrying a C-terminal 6×His tag (HHHHHH) and KDEL sequence. (B) SDS-PAGE of purified rBC2LCN and rBC2LCN-PE23. Four micrograms of purified rBC2LCN and rBC2LCN-PE23 in either the presence or absence of 2-mercaptoethanol (2ME) was run on a 5%–20% acrylamide gel and stained with Coomassie G-250. See also Figure S1 and Table S1.

We have demonstrated previously, by comprehensive glycome analysis using high-density lectin microarrays, that rBC2LCN binds specifically to hPSCs and not to somatic cells (Tateno et al., 2011). rBC2LCN (156 amino acids) was fused with a truncated form of catalytic domain of *P. aeruginosa* exotoxin A (399–613 residues; 215 amino acids) carrying a C-terminal 6×His tag (HHHHHH) and KDEL sequence via a ten amino acid linker (GSG_3_)_2_ (Figure 1A). The generated rBC2LCN-PE23 (396 amino acids) was expressed in *E. coli* and purified by one-step affinity chromatography using an L-fucose-Sepharose column. The yield was ∼10 mg/l of bacterial culture. rBC2LCN-PE23 gave a major band at the predicted molecular weight of 42 kDa on SDS-PAGE in either the presence or absence of 2-mercaptoethanol, whereas rBC2LCN gave a major band at 16 kDa (Figure 1B). Similar to rBC2LCN, rBC2LCN-PE23 was soluble and showed no tendency to aggregate, at least up to 3 months.

### Glycan-Binding Property of rBC2LCN-PE23

We analyzed the glycan-binding property of rBC2LCN-PE23 compared to rBC2LCN by glycoconjugate microarray (Tateno et al., 2008) and frontal affinity chromatography (Tateno et al., 2007). Based on glycoconjugate microarray analysis, rBC2LCN-PE23 exhibited a similar glycan-binding specificity to that of rBC2LCN; both bound to Fucα1-2Galβ1-3GlcNAc/GalNAc-containing glycans, such as Fucα1-2Galβ1-3GlcNAc (H type1), Fucα1-2Galβ1-3GalNAc (H type3), and Fucα1-2Galβ1-3(Fucα1-4)GlcNAc (Le^b^) (Figure S1; Table S1). The dissociation constants (*K*_d_) of rBC2LCN and rBC2LCN-PE23 with H type1-*para-*nitrophenol (*p*NP) and H type3-*p*NP were then determined by frontal affinity chromatography (Tateno et al., 2007). rBC2LCN-PE23 bound to H type1-*p*NP and H type3-*p*NP with *K*_d_ of 9.9 and 32.3 μM, respectively, similar to the dissociation constants of rBC2LCN to H type1-*p*NP (8.3 μM) and H type3-*p*NP (25.4 μM). These results demonstrate that rBC2LCN-PE23 exhibits glycan-binding properties similar to rBC2LCN.

### rBC2LCN-PE23 Is Internalized by hiPSCs

**Figure 2:**
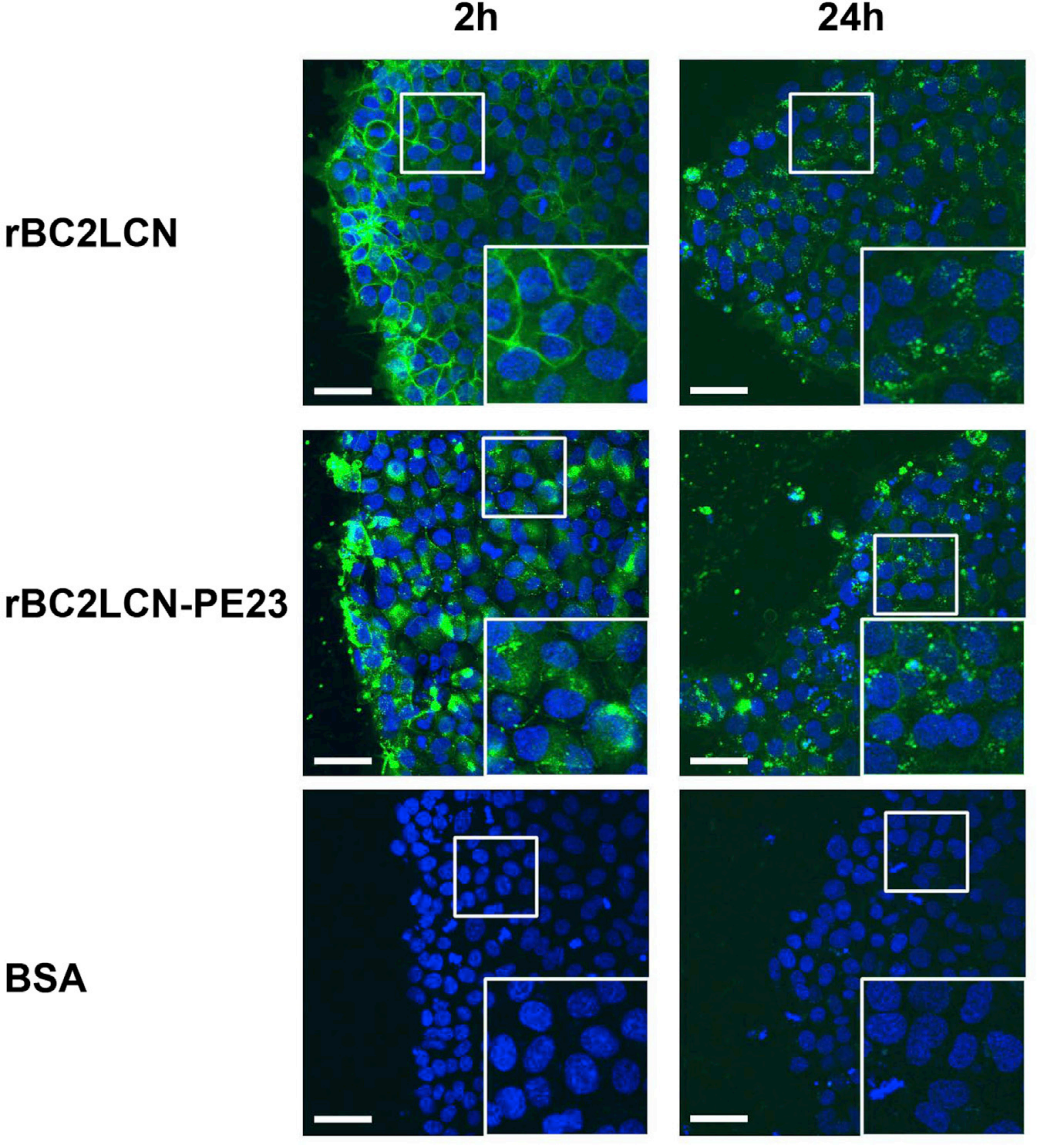
Internalization of rBC2LCN and rBC2LCN-PE23 The 201B7 hiPSCs were live-stained with either 1 μg/ml FITC-labeled rBC2LCN, FITC-labeled rBC2LCN-PE23, or FITC-labeled BSA for 2 hr. After replacing the medium without probes, the cells were cultured for 24 hr. Representative data are shown from two independent experiments. Scale bar, 50 μm.

We then examined whether rBC2LCN-PE23 binds to and is internalized by hiPSCs. FITC-labeled rBC2LCN or FITC-labeled rBC2LCN-PE23 (10 μg/ml) was added to the medium of 201B7 hiPSC cultures, which were incubated for 2 hr. As shown in Figure 2, FITC-rBC2LCN stained the cell membrane, whereas the cell membrane was only weakly stained with FITC-rBC2LCN-PE23 and most staining was already observed inside the cells. After replacing the medium without probes, the cells were cultured for a further 24 hr, after which punctate staining was clearly observed for both FITC-rBC2LCN and FITC-rBC2LCN-PE23, indicating that they were internalized by the cells. This observation strongly suggests that rBC2LCN could be used to deliver cytotoxic agents inside hPSCs.

### Cytotoxic Effect of rBC2LCN-PE23 on hPSCs

**Figure 3:**
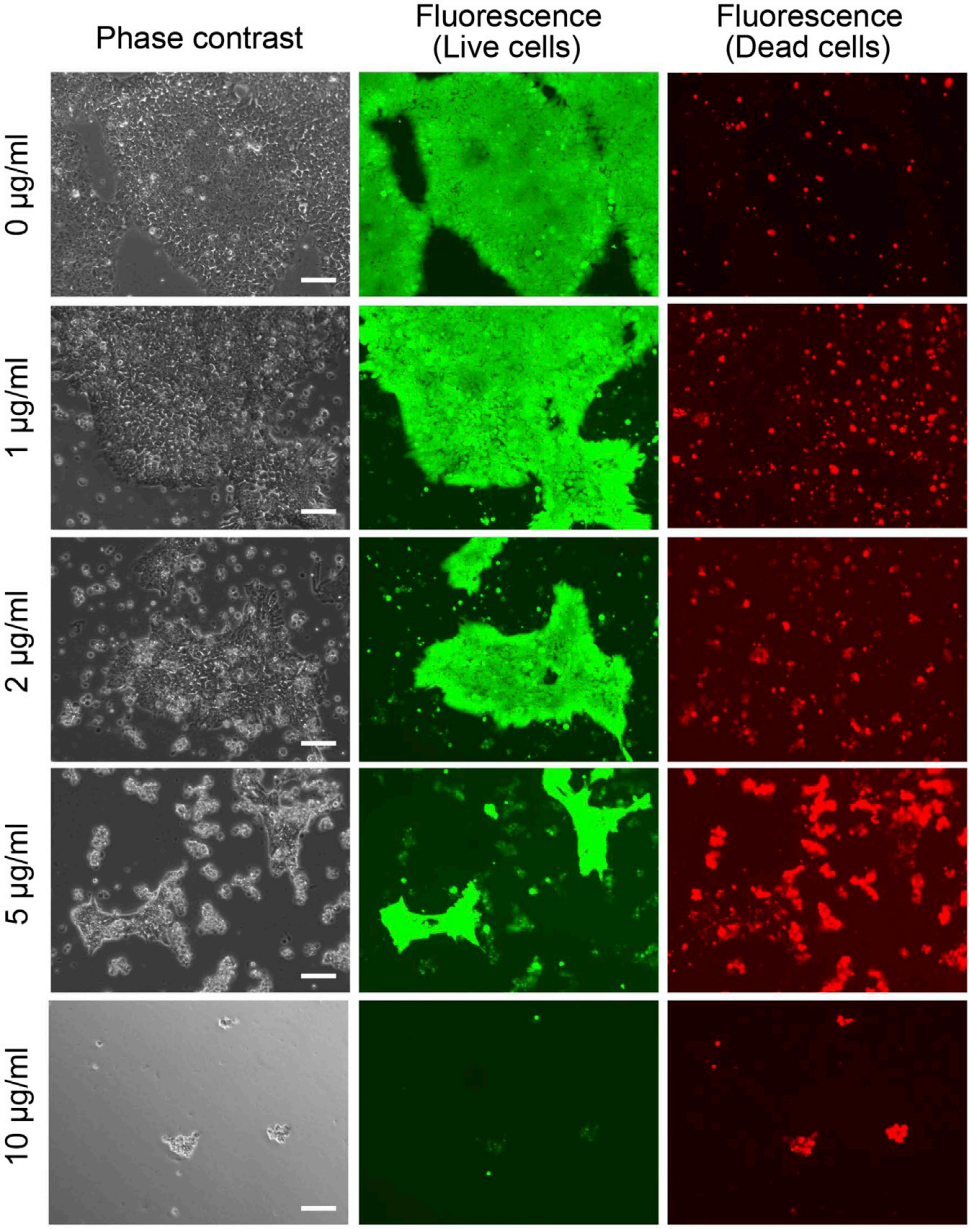
Effect of rBC2LCN-PE23 on the Cell Viability of hiPSCs The 201B7 hiPSCs were cultured in mTeSR1 containing 0, 1, 2, 5, or 10 μg/ml rBC2LCN-PE23. After 24 hr, the cells were stained and observed by fluorescence microscopy. Live cells stained cytoplasmic green fluorescence and dying or dead cells stained nuclear red fluorescence. Scale bar, 100 μm. See also Figures S2 and S3.

**Figure 4:**
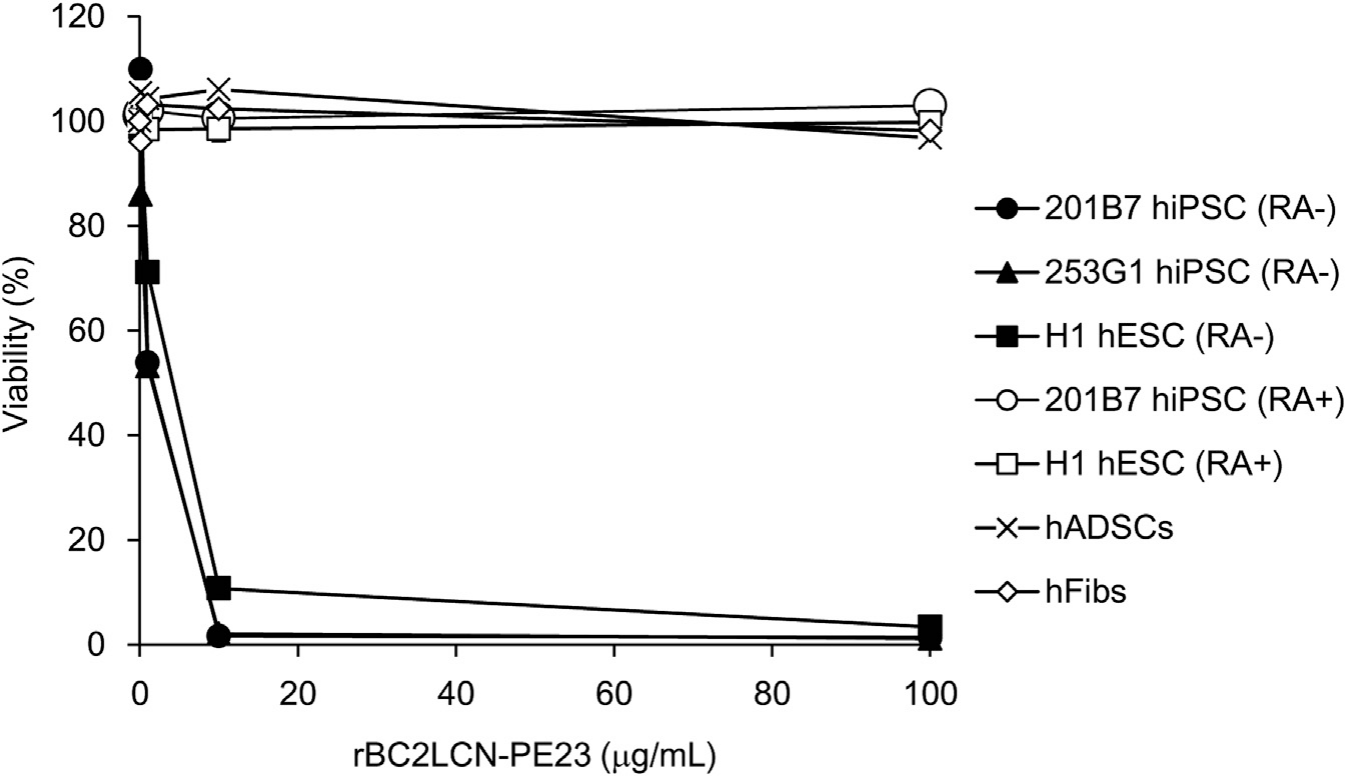
Quantitative Effect of rBC2LCN-PE23 on the Cell Viability Cells were cultured in either the presence or absence of rBC2LCN-PE23 (0.1, 1, 10, and 100 μg/ml) to determine its cytotoxicity. After 24 hr, the medium was replaced with fresh medium supplemented with 20 μl CCK-8 solution, and the absorbance at 450 nm was measured after a further 4-hr incubation. Viability (%) was calculated from the absorbance at 450 nm of treated cells relative to untreated cells. Data are shown as mean ± SD of triplicates. RA−, without RA treatment; RA+, with RA treatment.

Having demonstrated that rBC2LCN-PE23 is internalized by cells, we next examined whether it could induce cell death of hPSCs. 201B7 hiPSCs were cultured in the presence (1, 2, 5, and 10 μg/ml) or absence of rBC2LCN-PE23 (Figure 3). After 24 hr, the cells were stained using a live/dead cell imaging kit and observed under the microscope. With this reagent, live cells are stained with cytoplasmic green fluorescence, and dying and dead cells are stained with nuclear red fluorescence. In the absence of rBC2LCN-PE23, the green fluorescence-positive live cells proliferated normally and only a small number of dead cells stained red, demonstrating that most of the cells were viable. However, the number of green fluorescence-positive 201B7 hiPSCs decreased as the concentration of rBC2LCN-PE23 was increased. The green fluorescence-positive 201B7 hiPSCs disappeared when the cells were cultured in the presence of 10 μg/ml rBC2LCN-PE23 for 24 hr. Most of the dying or dead cells had detached from the cell culture flasks and were floating in the medium. The effect of rBC2LCN-PE23 also was quantified by measuring the metabolic activity of living cells. As shown in Figure 4, 201B7 hiPSCs were completely eliminated by 24 hr treatment with 10 μg/ml rBC2LCN-PE23. Similar results were obtained for 253G1 hiPSCs (Figures 4 and S2) and H1 hESCs (Figures 4 and S3). Taken together, these findings demonstrate that rBC2LCN-PE23 exerts a robust cytotoxic effect on hPSCs.

### Effect of rBC2LCN-PE23 on Differentiated Cells

**Figure 5:**
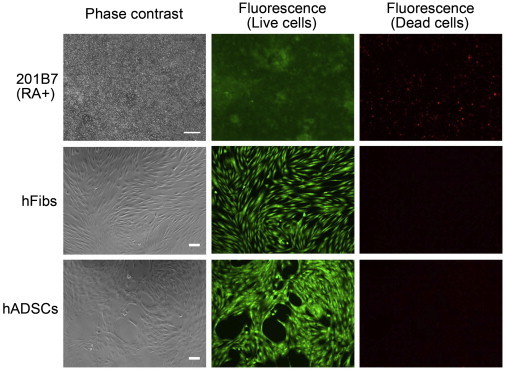
Effect of rBC2LCN-PE23 on the Cell Viability of Differentiated Cells The 201B7 hiPSCs were cultured in mTeSR1 containing 10 μM RA for 10 days. hFibs and hADSCs were cultured in mTeSR1 containing 10 μg/ml rBC2LCN-PE23. After 24 hr, cells were stained and observed by fluorescence microscopy. Live cells stained cytoplasmic green fluorescence and dying or dead cells stained nuclear red fluorescence. Representative data are shown from at least three independent experiments. Scale bar, 100 μm.

We then examined the sensitivity of 201B7 hiPSCs in response to rBC2LCN-PE23 after differentiation for 10 days with RA (Figure 5). Most of RA-treated 201B7 hiPSCs proliferated normally and stained with green fluorescence, indicative of viable cells, with only a small number of cells staining red, even after 24 hr treatment with 10 μg/ml rBC2LCN-PE23 (Figure 5, top). No effect of rBC2LCN-PE23 on the viability of RA-treated 201B7 hiPSCs was observed at concentrations up to 100 μg/ml (Figure 4).

We also analyzed the effect of rBC2LCN-PE23 on the viability of hFibs and hADSCs. Both hFibs and hADSCs were stained with green fluorescence, but not red fluorescence, even after 24 hr treatment with 10 μg/ml rBC2LCN-PE23 (Figure 5, middle and bottom), and substantially no quantitative effect on viability was observed (Figure 4). These results demonstrate that rBC2LCN-PE23 exerts a negligible cytotoxic effect on differentiated cells, such as RA-differentiated hiPSCs, hADSCs, and hFibs.

### Selective Elimination of hiPSCs in a Mixed Cell Population

**Figure 6:**
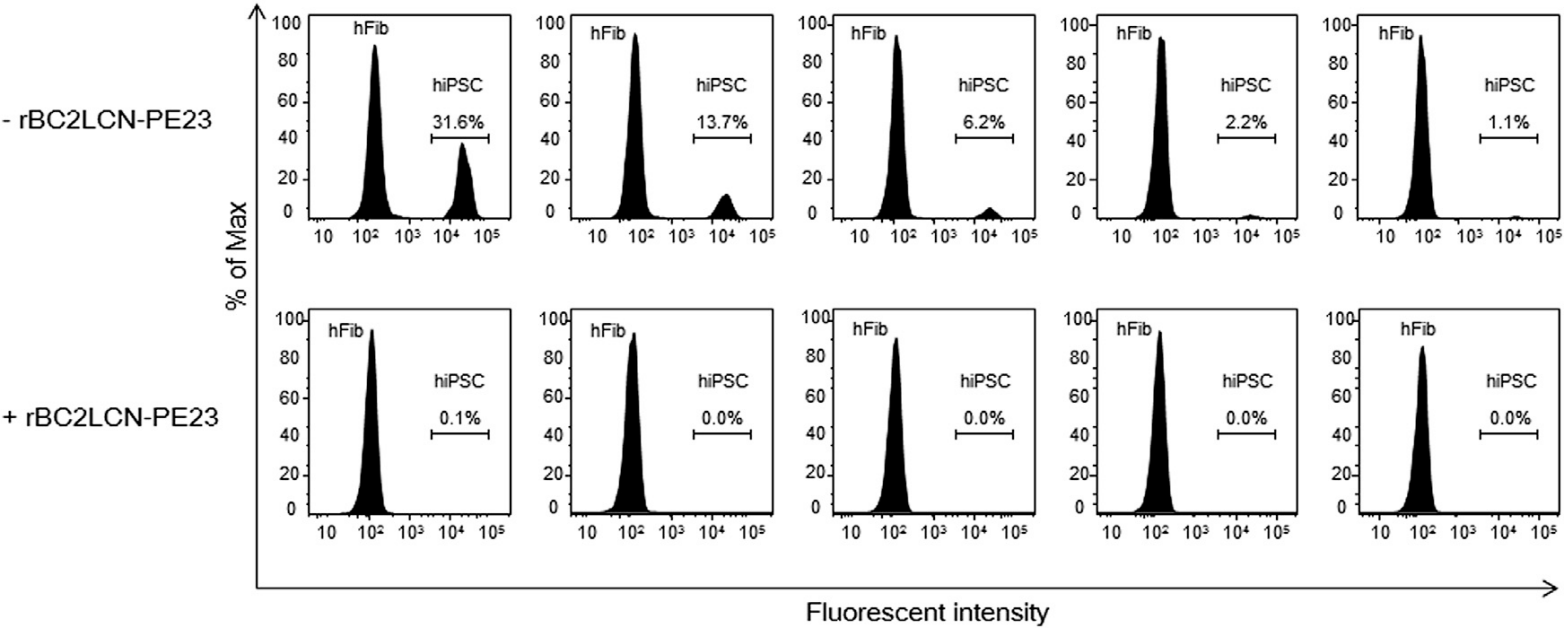
Selective Killing of hiPSCs by rBC2LCN-PE23 in a Mixed Cell Population Varying numbers of 201B7 hiPSCs were cultured and fluorescently labeled with 20 μM CellTracker Green CMFDA. After washing to remove residual fluorescence, hFibs were mixed and co-cultured in either the presence (bottom) or absence (top) of 10 μg/ml rBC2LCN-PE23. After 40 hr, flow cytometry data were acquired.

A varying number of 201B7 hiPSCs cultured on 12-well plates were labeled with CellTracker Green 5-chloromethylfluorescein diacetate (CMFDA). After removing residual fluorescence, fluorescently labeled 201B7 hiPSCs were co-cultured with hFibs in either the presence or absence of 10 μg/ml rBC2LCN-PE23. After 40 hr, cells were recovered and analyzed by flow cytometry (Figure 6). In the absence of rBC2LCN-PE23, fluorescently labeled 201B7 hiPSCs and unlabeled hFibs were detected in different ratios (31.6%–1.1% of 201B7 hiPSCs) (Figure 6, top). In the presence of rBC2LCN-PE23, however, fluorescently labeled 201B7 hiPSCs almost completely disappeared (Figure 6, bottom). These results demonstrate that rBC2LCN-PE23 can selectively eliminate hiPSCs even in a mixed cell population with differentiated cells at different ratios.

## Discussion

hPSCs are a leading candidate as a source of cells for regenerative medicine due to their capacity for self-renewal and pluripotency. Despite their therapeutic promise, a crucial hurdle for the clinical implementation of hPSC therapies is their tumorigenic potential. In this regard, various strategies to eliminate hPSCs have been reported. One of the major solutions involves antibody-based cell sorting. However, processing of cells in this way is time-consuming, expensive, and could affect cell viability. In addition, this method is not directly applicable to two-/three-dimensional cell cultures, such as cell sheets and tissues, requiring single-cell preparation prior to separation. Another popular solution is to use chemical inhibitors to induce the selective cell death of tumorigenic hPSCs. Given that chemical inhibitors are structurally defined, can be stably supplied in large amounts, and show no/minimal antigenicity, they are strong candidates to eliminate tumorigenic hPSCs. However, one of the major concerns with using small compounds is selectivity, i.e., possibility of side effect, which could affect the viability of differentiated cells and cause adverse effects, as differentiated cells share essentially the same metabolic system as hPSCs.

One of the key findings of this study is that rBC2LCN is internalized by hiPSCs. Taking advantage of this phenomenon, rBC2LCN was used as a cargo transporter to deliver *P. aeruginosa* exotoxin A, which inactivates protein synthesis. *P. aeruginosa* exotoxin A is synthesized as a single 638 residue polypeptide that is processed by the removal of 25 N-terminal residues before secretion as the 613 residue native toxin (Weldon and Pastan, 2011). It consists of three major structural domains. The N-terminal domain I is a receptor-binding domain, which is divided into two non-sequential domains, domain Ia (1–252 residues) and domain Ib (365–404 residues). Domain II (253–364 residues) has been proposed to be involved in toxin translocation and intracellular trafficking. Domain III (395–613 residues) is a catalytic domain, which catalyzes the inactivation of eukaryotic translation elongation factor 2 (eEF2), an essential factor for protein synthesis, by transferring an ADP-ribosyl group from NAD+ to the diphthamide residue (Weldon and Pastan, 2011). When fused with antibodies, *P. aeruginosa* exotoxin A has been used to create immunotoxins for the treatment of B cell malignancies, mesothelioma, lung cancer, and brain tumors, some of which have reached clinical trials (Weldon and Pastan, 2011). The most commonly used fragment of *P. aeruginosa* exotoxin A is a 38-kDa domain known as PE38, containing a deletion of the majority of domain Ia (Δ1–250 residues) and a portion of domain Ib (Δ365–380 residues) from the native form.

In this study, we fused 23 kDa of the catalytic domain (399–613 residues), which may be the smallest fragment of *P. aeruginosa* exotoxin A ever reported. The resulting recombinant cytotoxic lectin, rBC2LCN-PE23, could be expressed as a soluble form from the cytoplasm of *E. coli* and purified to homogeneity by one-step affinity chromatography at a relatively high yield (∼10 mg/l) sufficient for the industrial use. In addition, rBC2LCN-PE23 exhibited the same glycan-binding property as the native rBC2LCN. These results suggest that fusion with the catalytic domain of *P. aeruginosa* exotoxin A at the C terminus of rBC2LCN via an amino acid linker resulted in no/minimal effect on the native structure of the latter, which forms a homotrimer (Sulák et al., 2010).

Although the fate of rBC2LCN-PE23 after internalization has not been known, the chimeric protein is thought to basically undergo an intoxication pathway similar to that of *P. aeruginosa* exotoxin A (Weldon and Pastan, 2011). After internalization, *P. aeruginosa* exotoxin A is cleaved at a site in domain II by the endoprotease furin. In the Golgi, *P. aeruginosa* exotoxin A encounters KDEL receptors that transport the toxin to the ER. In the ER, the disulfide bond is reduced and the C-terminal domain containing the catalytic domain (domains II, Ib, and III) is released into the cytosol. In the cytosol, the C-terminal domain transfers an ADP-ribosyl group from NAD+ to the diphtamide residue of eEF2. This halts protein synthesis and leads to apoptotic cell death. We are now studying in detail the intracellular trafficking of rBC2LCN-PE23 using fluorescence microscopy and electron microscopy.

The addition of only 10 μg/ml rBC2LCN-PE23 to the culture medium was sufficient to completely eliminate hiPSCs after 24 hr, even in a mixed cell population. The dispersion of colonies was not a prerequisite to induce cell death of hiPSCs, demonstrating the high permeation ability of the developed reagent. rBC2LCN-PE23 was also effective in the case of hESCs, as rBC2LCN bound to all the types of hESCs (KhES-1/2/3, H1/7) tested (Onuma et al., 2013; Tateno et al., 2011). No effect was observed for RA-treated hiPSCs, hFibs, and ADSCs, in agreement with a previous report that rBC2LCN shows no binding to differentiated cells (Onuma et al., 2013; Tateno et al., 2011).

One possible concern could be the toxicity of residual rBC2LCN-PE23 when transplanting cells, although this could be washed away easily. Even if residual rBC2LCN-PE23 exists, there should be no effect on differentiated cells, since rBC2LCN-PE23 cannot bind to and be internalized by differentiated cells. Outside of the cells, *P. aeruginosa* exotoxin A exhibits no toxicity predicted from the intoxication pathway of the toxin.

The formation of neutralizing antibodies is a common occurrence when foreign proteins are used as therapeutic agents in humans. Given that rBC2LCN-PE23 containing rBC2LCN and the catalytic domain of *P. aeruginosa* exotoxin A is bacterial in origin, it is more likely that an immune response might be generated if rBC2LCN-PE23 is carried into humans. In this regard, the generation of less immunogenic rBC2LCN-PE23 might be important, and identifying and eliminating B cell and T cell epitopes from its sequence (King et al., 2014) should be considered.

As described herein, rBC2LCN-PE23 could be stably supplied and is cost-effective, and should be applicable to various experiments requiring the elimination of hPSCs, including the generation of hPSC-derived cells for drug screening as well as therapy. For application of rBC2LCN-PE23 to hPSC-based therapy, an appropriate protocol would need to be developed in each clinical setting in terms of standard operating procedure (SOP). hiPSC-derived cardiomyocytes require approximately 10^8^–10^9^ cells for implantation to produce cardiac improvements (Kawamura et al., 2012, 2013). In this case, even 1% of hiPSCs corresponds to 10^6^–10^7^ cells, which is far more than enough for teratoma formation (Gropp et al., 2012; Hentze et al., 2009), which may cause life-threatening events such as fatal arrhythmias, cardiac tamponade, and heart failure (Yoshida and Yamanaka, 2010). Therefore, it is mandatory to eliminate the risk of teratoma formation to realize the safety and efficacy of hiPSC-derived cardiomyocytes.

## Experimental Procedures

### Construction, Expression, and Purification of rBC2LCN-PE23

An N-terminal ten amino acid linker (GSG_3_)_2_, the truncated form of the catalytic domain of *P. aeruginosa* exotoxin A (399–613 amino acid, 23 kDa), and a C-terminal 6×His tag (PPHHHHHH) followed by Lys-Asp-Glu-Leu (KDEL) was inserted via *NheI* and *XhoI* into an expression vector of pET27b containing an N-terminal domain (1–156 amino acid) of BC2L-C identified from *Burkholderia cenocepacia* (rBC2LCN). The total number of amino acids was 396 and the calculated average molecular weight was 41,673 Da. The generated rBC2LCN-toxin fusion protein was designated as rBC2LCN-PE23.

The expression plasmid of rBC2LCN-PE23 was transformed into *E. coli* BL21 CodonPlus (DE3)-RIL. The transformed *E. coli* was cultured in LB medium containing 10 μg/ml kanamycin at 37°C until the OD600 reached 0.4. Expression of rBC2LCN-PE23 was induced by the addition of 1 mM IPTG at 20°C for 24 hr. The following procedures were performed at 4°C. The *E. coli* cells were harvested by centrifugation at 4,450 × *g* for 30 min and lyzed in PBSET (6 mM Na_2_HPO_4_・12H_2_O, 1.4 mM KH_2_PO_4_, 140 mM NaCl [pH 7.0], 1 mM EDTA, and 0.1% Triton X-100) containing protease inhibitor cocktail (Nacalai tesque) by sonication. After centrifugation at 24,910 × *g* for 30 min, supernatants were applied onto L-fucose-Sepharose and the bound rBC2LCN-PE23 was eluted with 0.2 M L-fucose in PBSE (6 mM Na_2_HPO_4_・12H_2_O, 1.4 mM KH_2_PO_4_, 140 mM NaCl [pH 7.0], and 1 mM EDTA). The purified rBC2LCN-PE23 was dialyzed against PBS. The protein concentration was measured by BCA protein assay (Thermo Scientific) and the purity was analyzed by electrophoresis using 17% XV pantera MP Gel (DRC).

### SDS-PAGE

Four micrograms of purified rBC2LCN or rBC2LCN-PE23 was incubated at 95°C for 5 min in either the presence or absence of 2-mercaptoethanol and run on a 5%–20% polyacrylamide gel (DRC). The gel was then stained with Bio-Safe Coomassie G-250 Stain (Bio-Rad).

### Frontal Affinity Chromatography

The principle and protocol for frontal affinity chromatography have been described previously (Nakamura et al., 2005; Tateno et al., 2007).

### Cell Culture

The 201B7 and 253G1 hiPSCs were cultured in mTeSR1 (STEMCELL Technologies) on cell culture plates coated with Matrigel (BD Biosciences) (Nakagawa et al., 2008; Takahashi et al., 2007). The hESC line H1 was cultured in mTeSR1 according to the WiCell Feeder Independent Pluripotent Stem Cell Protocols provided by the WiCell Research Institute (http://www.wicell.org). 201B7 and H1 hESCs were differentiated by culturing them in mTeSR1 containing 10 μM RA for 10 days. hFibs and hADSCs were cultured in MesenPRO RS medium. Cells were counted with a hemocytometer, a Vi-CELL Cell Viability Analyzer (Beckman Coulter Genomics), or a TC20 Automated Cell Counter (Bio-Rad). hiPSCs and hESCs used in this study were confirmed to have the ability to form teratoma and be positive for both anti-SSEA4 and rBC2LCN (Tateno et al., 2014).

### Internalization Assay

201B7 hiPSCs were cultured in mTeSR1 on four-well coverglass chambers (AGC Techno Glass) coated with Matrigel. Hoechist33342 (Dojindo) (0.5 μg/ml) and 1 μg/ml of either FITC-labeled rBC2LCN, FITC-labeled rBC2LCN-PE23, or FITC-labeled BSA were added to the culture medium for 2 hr. After replacing the medium without probes, the cells were observed under a FluoView FV1000 confocal laser-scanning microscope (Olympus).

### Cell Viability Assay

The cytotoxic effect of rBC2LCN-PE23 was analyzed using a LIVE/DEAD Cell Imaging Kit (Molecular Probes). Cells were cultured in mTeSR1 on 6- or 12-well plates coated with Matrigel in the presence or absence of rBC2LCN-PE23 (1 and 10 μg/ml). After 24 hr, they were stained with Live Green (A) and Dead Red (B) reagents, according to the manufacturer’s instructions, and observed under an Axio Vert.A1 (Carl Zeiss) or BZ-9000 fluorescence microscope.

The cytotoxic effect of rBC2LCN-PE23 also was analyzed using a Cell Counting Kit-8 (Dojindo). Cells were cultured in 500 μl mTeSR1 on 24-well plates coated with Matrigel in the presence or absence of rBC2LCN-PE23 (0.1, 1, 10, and 100 μg/ml). After 24 hr, the culture medium was replaced with 200 μl fresh medium and 20 μl CCK-8 solution, and, after a further 4-hr incubation in a CO_2_ incubator, the absorbance at 450 nm was measured.

### Flow Cytometry

Varying numbers of 201B7 hiPSCs were cultured in mTeSR1 on 12-well plates coated with Matrigel. Adherent 201B7 hiPSCs were fluorescently labeled with 20 μM CellTracker Green CMFDA (Life Technologies) at 37°C for 45 min. After replacing the medium, cells were further cultured at 37°C for 30 min. Unlabeled hFibs were seeded and co-cultured in either the presence or absence of 10 μg/ml rBC2LCN-PE23. After 40 hr, cells were recovered and centrifuged at 5,000 rpm for 1 min. After removing the supernatant, the cells were suspended in 1 ml PBS containing 1% BSA. Finally, flow cytometry data were acquired on a FACSCanto-II cytometer (BD Biosciences) and analyzed using FlowJo software (Tree Star).

## Author Contributions

H.T., Y.O., and Y.I. designed the research. H.T., Y.O., Y.I., F.M., S.S., M.S., and Y.A. performed the research. H.T., Y.O., and Y.I. analyzed the data. H.T. wrote the paper. J.H. and M.A. supervised the research.

## Supplemental Information

Document S1. Figures S1–S3 and Table S1

Document S2. Article plus Supplemental Information

## References

[bib1] Ben-David U., Benvenisty N. (2011). The tumorigenicity of human embryonic and induced pluripotent stem cells. Nat. Rev. Cancer.

[bib2] Ben-David U., Gan Q.F., Golan-Lev T., Arora P., Yanuka O., Oren Y.S., Leikin-Frenkel A., Graf M., Garippa R., Boehringer M. (2013). Selective elimination of human pluripotent stem cells by an oleate synthesis inhibitor discovered in a high-throughput screen. Cell Stem Cell.

[bib3] Ben-David U., Nudel N., Benvenisty N. (2013). Immunologic and chemical targeting of the tight-junction protein Claudin-6 eliminates tumorigenic human pluripotent stem cells. Nat. Commun..

[bib4] Choo A.B., Tan H.L., Ang S.N., Fong W.J., Chin A., Lo J., Zheng L., Hentze H., Philp R.J., Oh S.K., Yap M. (2008). Selection against undifferentiated human embryonic stem cells by a cytotoxic antibody recognizing podocalyxin-like protein-1. Stem Cells.

[bib5] Dam T.K., Brewer C.F. (2010). Maintenance of cell surface glycan density by lectin-glycan interactions: a homeostatic and innate immune regulatory mechanism. Glycobiology.

[bib6] Dam T.K., Gerken T.A., Brewer C.F. (2009). Thermodynamics of multivalent carbohydrate-lectin cross-linking interactions: importance of entropy in the bind and jump mechanism. Biochemistry.

[bib7] Garber K. (2013). Inducing translation. Nat. Biotechnol..

[bib8] Goldring C.E., Duffy P.A., Benvenisty N., Andrews P.W., Ben-David U., Eakins R., French N., Hanley N.A., Kelly L., Kitteringham N.R. (2011). Assessing the safety of stem cell therapeutics. Cell Stem Cell.

[bib9] Gropp M., Shilo V., Vainer G., Gov M., Gil Y., Khaner H., Matzrafi L., Idelson M., Kopolovic J., Zak N.B., Reubinoff B.E. (2012). Standardization of the teratoma assay for analysis of pluripotency of human ES cells and biosafety of their differentiated progeny. PLoS ONE.

[bib10] Hasehira K., Tateno H., Onuma Y., Ito Y., Asashima M., Hirabayashi J. (2012). Structural and quantitative evidence for dynamic glycome shift on production of induced pluripotent stem cells. Mol. Cell. Proteomics.

[bib11] Hentze H., Soong P.L., Wang S.T., Phillips B.W., Putti T.C., Dunn N.R. (2009). Teratoma formation by human embryonic stem cells: evaluation of essential parameters for future safety studies. Stem Cell Res. (Amst.).

[bib12] Kamao H., Mandai M., Okamoto S., Sakai N., Suga A., Sugita S., Kiryu J., Takahashi M. (2014). Characterization of human induced pluripotent stem cell-derived retinal pigment epithelium cell sheets aiming for clinical application. Stem Cell Reports.

[bib13] Kawai H., Yamashita T., Ohta Y., Deguchi K., Nagotani S., Zhang X., Ikeda Y., Matsuura T., Abe K. (2010). Tridermal tumorigenesis of induced pluripotent stem cells transplanted in ischemic brain. J. Cereb. Blood Flow Metab..

[bib14] Kawamura M., Miyagawa S., Miki K., Saito A., Fukushima S., Higuchi T., Kawamura T., Kuratani T., Daimon T., Shimizu T. (2012). Feasibility, safety, and therapeutic efficacy of human induced pluripotent stem cell-derived cardiomyocyte sheets in a porcine ischemic cardiomyopathy model. Circulation.

[bib15] Kawamura M., Miyagawa S., Fukushima S., Saito A., Miki K., Ito E., Sougawa N., Kawamura T., Daimon T., Shimizu T. (2013). Enhanced survival of transplanted human induced pluripotent stem cell-derived cardiomyocytes by the combination of cell sheets with the pedicled omental flap technique in a porcine heart. Circulation.

[bib16] Kershaw D.B., Beck S.G., Wharram B.L., Wiggins J.E., Goyal M., Thomas P.E., Wiggins R.C. (1997). Molecular cloning and characterization of human podocalyxin-like protein. Orthologous relationship to rabbit PCLP1 and rat podocalyxin. J. Biol. Chem..

[bib17] King C., Garza E.N., Mazor R., Linehan J.L., Pastan I., Pepper M., Baker D. (2014). Removing T-cell epitopes with computational protein design. Proc. Natl. Acad. Sci. USA.

[bib18] Lee A.S., Tang C., Cao F., Xie X., van der Bogt K., Hwang A., Connolly A.J., Robbins R.C., Wu J.C. (2009). Effects of cell number on teratoma formation by human embryonic stem cells. Cell cycle.

[bib19] Lee A.S., Tang C., Rao M.S., Weissman I.L., Wu J.C. (2013). Tumorigenicity as a clinical hurdle for pluripotent stem cell therapies. Nat. Med..

[bib20] Lee M.O., Moon S.H., Jeong H.C., Yi J.Y., Lee T.H., Shim S.H., Rhee Y.H., Lee S.H., Oh S.J., Lee M.Y. (2013). Inhibition of pluripotent stem cell-derived teratoma formation by small molecules. Proc. Natl. Acad. Sci. USA.

[bib21] Nakagawa M., Koyanagi M., Tanabe K., Takahashi K., Ichisaka T., Aoi T., Okita K., Mochiduki Y., Takizawa N., Yamanaka S. (2008). Generation of induced pluripotent stem cells without Myc from mouse and human fibroblasts. Nat. Biotechnol..

[bib22] Nakamura S., Yagi F., Totani K., Ito Y., Hirabayashi J. (2005). Comparative analysis of carbohydrate-binding properties of two tandem repeat-type Jacalin-related lectins, Castanea crenata agglutinin and Cycas revoluta leaf lectin. FEBS J..

[bib23] Onuma Y., Tateno H., Hirabayashi J., Ito Y., Asashima M. (2013). rBC2LCN, a new probe for live cell imaging of human pluripotent stem cells. Biochem. Biophys. Res. Commun..

[bib24] Richards M., Phoon C.W., Goh G.T., Seng E.K., Guo X.M., Tan C.M., Chan W.K., Lee J.M. (2014). A new class of pluripotent stem cell cytotoxic small molecules. PLoS ONE.

[bib25] Roy N.S., Cleren C., Singh S.K., Yang L., Beal M.F., Goldman S.A. (2006). Functional engraftment of human ES cell-derived dopaminergic neurons enriched by coculture with telomerase-immortalized midbrain astrocytes. Nat. Med..

[bib26] Schuldiner M., Itskovitz-Eldor J., Benvenisty N. (2003). Selective ablation of human embryonic stem cells expressing a “suicide” gene. Stem Cells.

[bib27] Sulák O., Cioci G., Delia M., Lahmann M., Varrot A., Imberty A., Wimmerová M. (2010). A TNF-like trimeric lectin domain from Burkholderia cenocepacia with specificity for fucosylated human histo-blood group antigens. Structure.

[bib28] Takahashi K., Tanabe K., Ohnuki M., Narita M., Ichisaka T., Tomoda K., Yamanaka S. (2007). Induction of pluripotent stem cells from adult human fibroblasts by defined factors. Cell.

[bib29] Tan H.L., Fong W.J., Lee E.H., Yap M., Choo A. (2009). mAb 84, a cytotoxic antibody that kills undifferentiated human embryonic stem cells via oncosis. Stem Cells.

[bib30] Tang C., Lee A.S., Volkmer J.P., Sahoo D., Nag D., Mosley A.R., Inlay M.A., Ardehali R., Chavez S.L., Pera R.R. (2011). An antibody against SSEA-5 glycan on human pluripotent stem cells enables removal of teratoma-forming cells. Nat. Biotechnol..

[bib31] Tateno H., Nakamura-Tsuruta S., Hirabayashi J. (2007). Frontal affinity chromatography: sugar-protein interactions. Nat. Protoc..

[bib32] Tateno H., Mori A., Uchiyama N., Yabe R., Iwaki J., Shikanai T., Angata T., Narimatsu H., Hirabayashi J. (2008). Glycoconjugate microarray based on an evanescent-field fluorescence-assisted detection principle for investigation of glycan-binding proteins. Glycobiology.

[bib33] Tateno H., Toyota M., Saito S., Onuma Y., Ito Y., Hiemori K., Fukumura M., Matsushima A., Nakanishi M., Ohnuma K. (2011). Glycome diagnosis of human induced pluripotent stem cells using lectin microarray. J. Biol. Chem..

[bib34] Tateno H., Matsushima A., Hiemori K., Onuma Y., Ito Y., Hasehira K., Nishimura K., Ohtaka M., Takayasu S., Nakanishi M. (2013). Podocalyxin is a glycoprotein ligand of the human pluripotent stem cell-specific probe rBC2LCN. Stem Cells Transl. Med..

[bib35] Tateno H., Onuma Y., Ito Y., Hiemori K., Aiki Y., Shimizu M., Higuchi K., Fukuda M., Warashina M., Honda S. (2014). A medium hyperglycosylated podocalyxin enables noninvasive and quantitative detection of tumorigenic human pluripotent stem cells. Sci. Rep..

[bib36] Thomson J.A., Itskovitz-Eldor J., Shapiro S.S., Waknitz M.A., Swiergiel J.J., Marshall V.S., Jones J.M. (1998). Embryonic stem cell lines derived from human blastocysts. Science.

[bib37] Tohyama S., Hattori F., Sano M., Hishiki T., Nagahata Y., Matsuura T., Hashimoto H., Suzuki T., Yamashita H., Satoh Y. (2013). Distinct metabolic flow enables large-scale purification of mouse and human pluripotent stem cell-derived cardiomyocytes. Cell Stem Cell.

[bib38] Vazquez-Martin A., Cufi S., Lopez-Bonet E., Corominas-Faja B., Oliveras-Ferraros C., Martin-Castillo B., Menendez J.A. (2012). Metformin limits the tumourigenicity of iPS cells without affecting their pluripotency. Sci. Rep..

[bib39] Wang Y.C., Nakagawa M., Garitaonandia I., Slavin I., Altun G., Lacharite R.M., Nazor K.L., Tran H.T., Lynch C.L., Leonardo T.R. (2011). Specific lectin biomarkers for isolation of human pluripotent stem cells identified through array-based glycomic analysis. Cell Res..

[bib40] Weldon J.E., Pastan I. (2011). A guide to taming a toxin—recombinant immunotoxins constructed from Pseudomonas exotoxin A for the treatment of cancer. FEBS J..

[bib41] Yamashita T., Kawai H., Tian F., Ohta Y., Abe K. (2011). Tumorigenic development of induced pluripotent stem cells in ischemic mouse brain. Cell Transplant..

[bib42] Yoshida Y., Yamanaka S. (2010). Recent stem cell advances: induced pluripotent stem cells for disease modeling and stem cell-based regeneration. Circulation.

