# Supplementary material for: Elimination of Tumorigenic Human Pluripotent Stem Cells by a Recombinant Lectin-Toxin Fusion Protein
Source: Stem Cell Reports. 2015 Apr 9;4(5):811–20. doi: 10.1016/j.stemcr.2015.02.016 (PMC4437484; doi:10.1016/j.stemcr.2015.02.016)
Supplement: Document S1. Figures S1–S3 and Table S1 [file mmc1.pdf]

Stem Cell Reports

Supplemental Information

Elimination of Tumorigenic Human Pluripotent Stem Cells by a Recombinant Lectin-Toxin Fusion Protein

Hiroaki Tateno, Yasuko Onuma, Yuzuru Ito, Fumi Minoshima, Sayoko Saito, Madoka Shimizu, Yasuhiko Aiki, Makoto Asashima, and Jun Hirabayashi

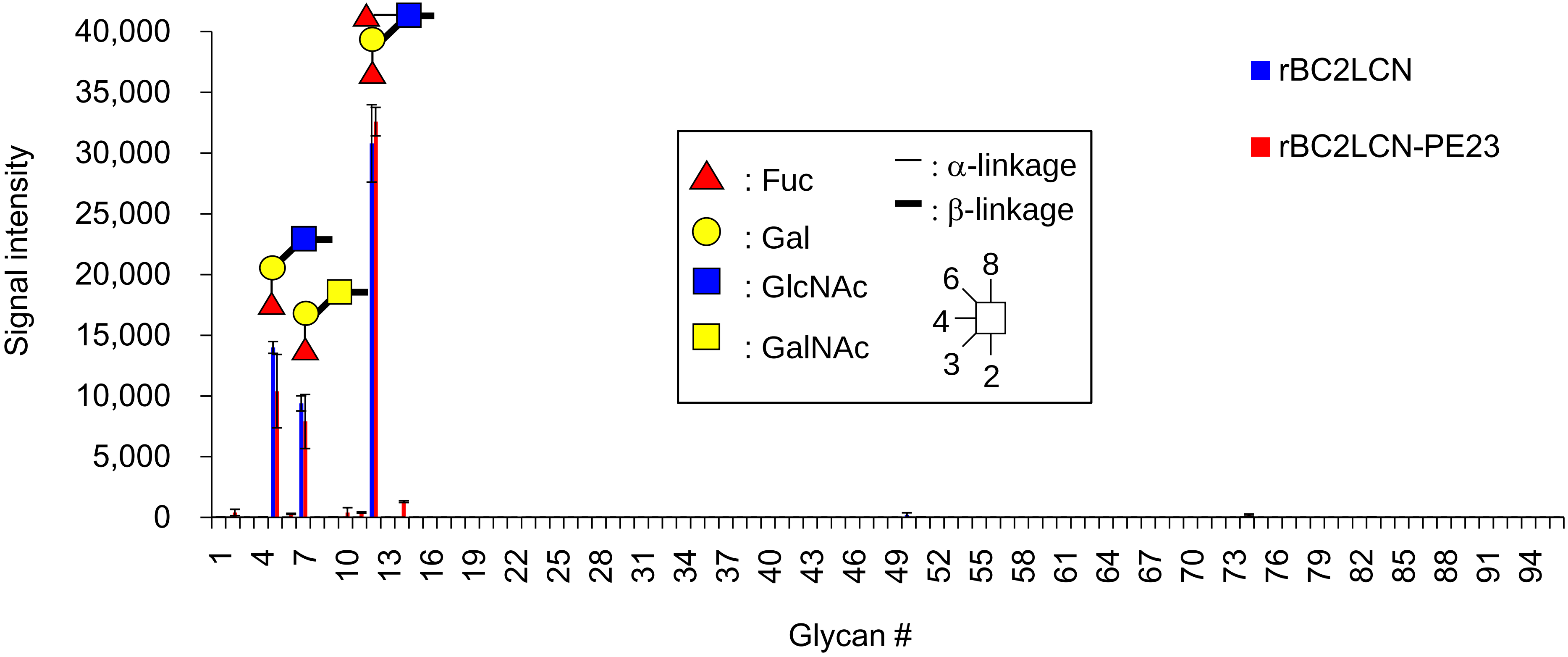

Figure S1, related to Figure 1 Glycoconjugate microarray analysis of rBC2LCN and rBC2LCN-PE23. Cy3-labeled rBC2LCN and Cy3-labeled rBC2LCN-PE23 were reacted with glycoconjugate microarray at 0.125 μg/mL overnight and scanned with an evanescent-field fluorescence scanner, GlycoStation Reader 1200. The fluorescence signal of each spot was quantified using Array Pro Analyzer ver.4.5, and the background value was subtracted. The signals of triplicate spots were averaged and shown as the average±S.D. Representative data are shown of two independent experiments.

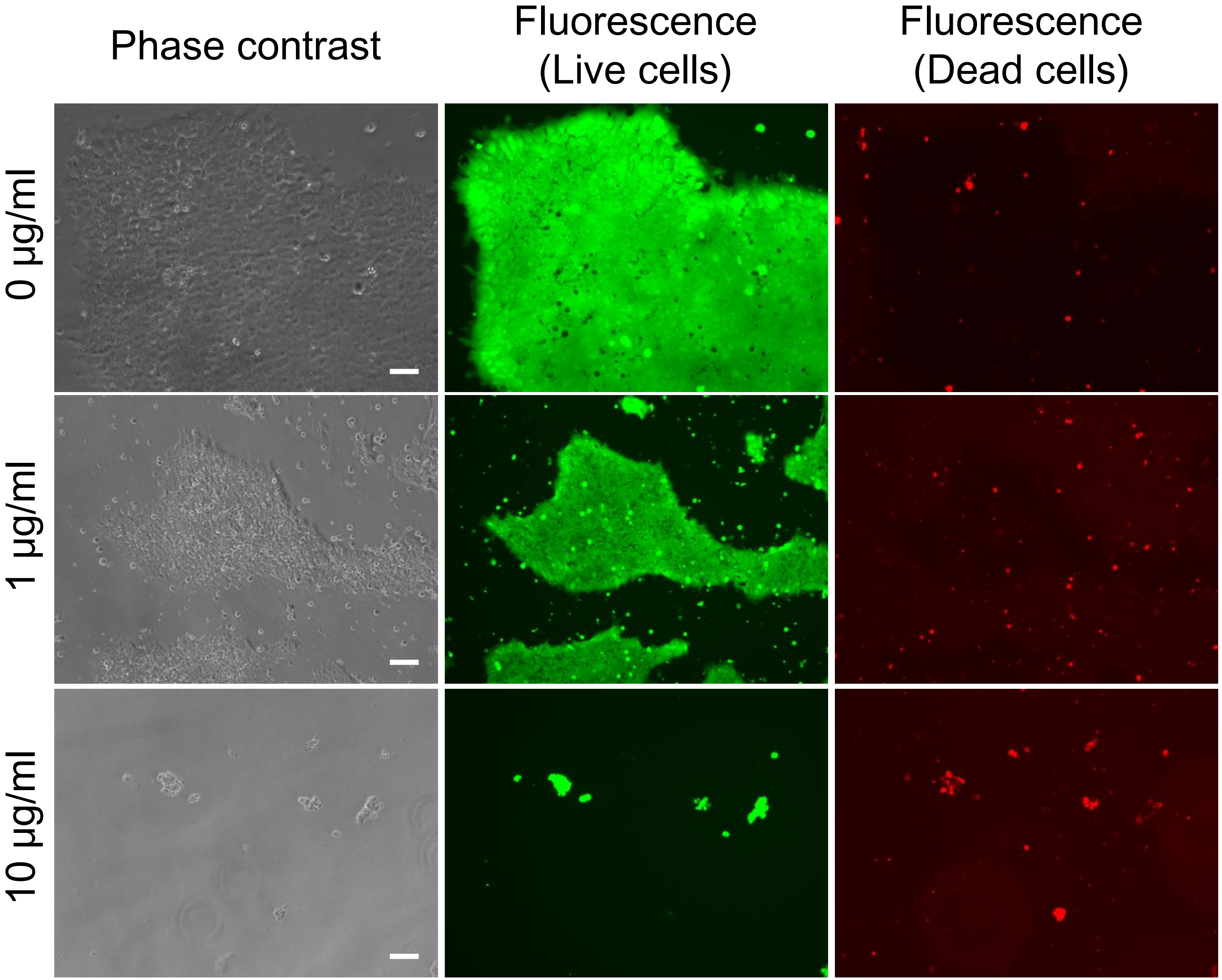

Figure S2, related to Figure 3. Effect of rBC2LCN-PE23 on 253G1 hiPSCs. 253G1 hiPSCs were cultured in mTeSR1 medium containing 0, 1, 10 μg/mL of rBC2LCN-PE23. After 24 h, cells were stained with LIVE/DEAD Cell Imaging Kit (Molecular Probes) and observed under fluorescence microscope. Live cells were stained in green fluorescence and dying or dead cells were stained in red fluorescence. Representative data are shown of two independent experiments. Scale bar: 100 μm.

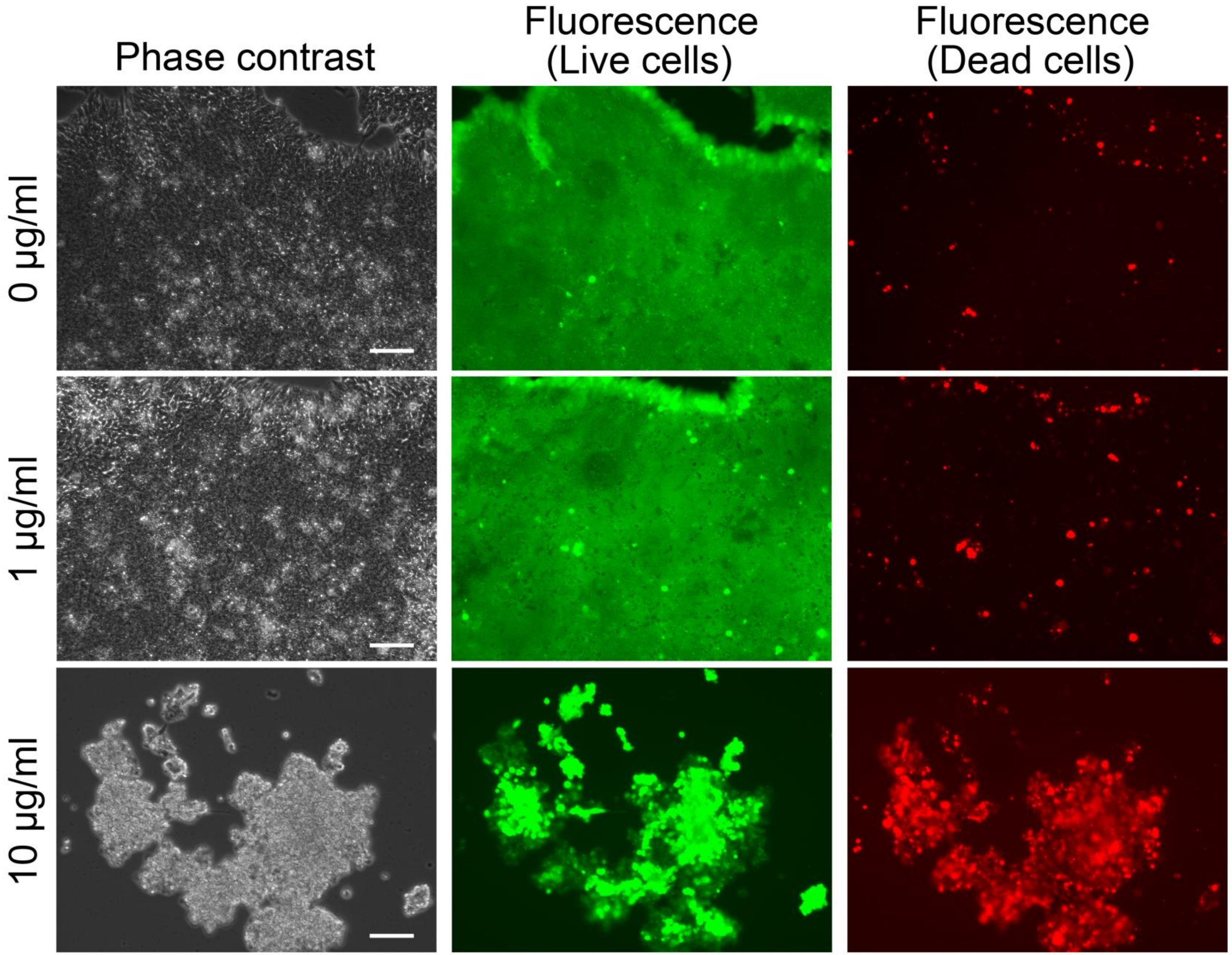

Figure S3, related to Figure 3. Effect of rBC2LCN-PE23 on hESCs H1. hESCs H1 cells were cultured in mTeSR1 medium containing 0, 1, 10 μg/mL of rBC2LCN-PE23. After 24 h, cells were stained with LIVE/DEAD Cell Imaging Kit (Molecular Probes) and observed under fluorescence microscope. Live cells were stained in green fluorescence and dying or dead cells were stained in red fluorescence. Representative data are shown of two independent experiments. Scale bar: 100 μm.

Table S1, related to Figure 1. Glycans used for glycoconjugate microarray.

Number	Trivial name	Presentation	Glycans	Co.	Cat#
1	αFuc	PAA	Fucα1-PAA	Glycotech	01-007
2	Fucα2Gal	PAA	Fucα1-2Galβ1-PAA	Glycotech	01-019
3	Fucα3GlcNAc	PAA	Fucα1-3GlcNAcβ1-PAA	Glycotech	01-024
4	Fucα4GlcNAc	PAA	Fucα1-4GlcNAcβ1-PAA	Glycotech	01-025
5	H type1	PAA	Fucα1-2Galβ1-3GlcNAcβ1-PAA	Glycotech	01-037
6	H type2	PAA	Fucα1-2Galβ1-4GlcNAcβ1-PAA	Glycotech	08-034
7	H type3	PAA	Fucα1-2Galβ1-3GalNAcα1-PAA	Glycotech	08-060
8	A	PAA	GalNAcα1-3(Fucα1-2)Galβ1-4GlcNAcβ1-PAA	Glycotech	08-091
9	B	PAA	Galα1-3(Fucα1-2)Galβ1-4GlcNAcβ1-PAA	Glycotech	08-092
10	Le^a	PAA	Galβ1-3(Fucα1-4)GlcNAcβ1-PAA	Glycotech	01-035
11	[3S]Le^a	PAA	(3OSO$_3$)Galβ1-3(Fucα1-4)GlcNAcβ1-PAA	Glycotech	01-040
12	Le^b	PAA	Fucα1-2Galβ1-3(Fucα1-4)GlcNAcβ1-PAA	Glycotech	08-042
13	Le^x	PAA	Galβ1-4(Fucα1-3)GlcNAcβ1-PAA	Glycotech	01-036
14	Le^y	PAA	Fucα1-2Galβ1-4(Fucα1-3)GlcNAcβ1-PAA	Glycotech	08-043
15	αNeu5Ac	PAA	Neu5Acα2-PAA	Glycotech	01-012
16	αNeu5Gc	PAA	Neu5Gcα2-PAA	Glycotech	01-051
17	Sia2	PAA	Neu5Acα2-8Neu5Acα2-PAA	Glycotech	08-064
18	Sia3	PAA	Neu5Acα2-8Neu5Acα2-8Neu5Acα2-PAA	Glycotech	01-081
19	3'SiaLe^c	PAA	Neu5Acα2-3Galβ1-3GlcNAcβ1-PAA	Glycotech	01-078
20	3'SL	PAA	Neu5Acα2-3Galβ1-4Glcβ1-PAA	Glycotech	01-038
21	3'SLN	PAA	Neu5Acα2-3Galβ1-4GlcNAcβ1-PAA	Glycotech	01-077
22	sLe^a	PAA	Neu5Acα2-3Galβ1-3(Fucα1-4)GlcNAcβ1-PAA	Glycotech	08-044
23	sLe^x	PAA	Neu5Acα2-3Galβ1-4(Fucα1-3)GlcNAcβ1-PAA	Glycotech	01-045
24	6'SL	PAA	Neu5Acα2-6Galβ1-4Glcβ1-PAA	Glycotech	01-039
25	FET	Glycoprotein	Fetuin (Complex-type N-glycans and O-glycans)	Sigma	F3004
26	AGP	Glycoprotein	α1-acid glycoprotein (Complex-type N-glycans)	Sigma	G9885
27	TF	Glycoprotein	Transferrin (Complex-type N-glycans)	Sigma	T3309
28	TG	Glycoprotein	Porcine thyroglobulin (Complex and high-mannnose-type	Sigma	T1126
29	βGal	PAA	Galβ1-PAA	Glycotech	01-004
30	[3S]βGal	PAA	(3OSO$_3$)Galβ1-PAA	Glycotech	01-015
31	A-di	PAA	GalNAcα1-3Galβ1-PAA	Glycotech	01-017
32	Lac	PAA	Galβ1-4Glcβ1-PAA	Glycotech	01-021
33	Le^c	PAA	Galβ1-3GlcNAcβ1-PAA	Glycotech	01-020
34	[3'S]Le^c	PAA	(3OSO$_3$)Galβ1-3GlcNAcβ1-PAA	Glycotech	01-062
35	LN	PAA	Galβ1-4GlcNAcβ1-PAA	Glycotech	01-022
36	[3'S]LN	PAA	(3OSO$_3$)Galβ1-4GlcNAcβ1-PAA	Glycotech	01-061
37	[6S]LN	PAA	Galβ1-4(6OSO$_3$)GlcNAcβ1-PAA	Glycotech	01-066
38	[6'S]LN	PAA	(6OSO$_3$)Galβ1-4GlcNAcβ1-PAA	Glycotech	01-068
39	βGalNAc	PAA	GalNAcβ1-PAA	Glycotech	01-011
40	di-GalNAcβ	PAA	GalNAcβ1-3GalNAcβ1-PAA	Glycotech	01-070
41	LDN	PAA	GalNAcβ1-4GlcNAcβ1-PAA	Glycotech	01-057
42	GA2	PAA	GalNAcβ1-4Galβ1-4Glcβ1-PAA	Glycotech	08-074
43	Asialo-FET	Glycoprotein	Asialo fetuin (Desialylated complex-type N- and O-glycans)	Sigma	F3004 (Acid-
44	Asialo-AGP	Glycoprotein	Asialo α1-acid glycoprotein (Desialylated complex-type N-	Sigma	G9885 (Acid-
45	Asialo-TF	Glycoprotein	Asialo transferrin (Desialylated complex-type N-glycans)	Sigma	T3309 (Acid-
46	Asialo-TG	Glycoprotein	Asialo porcine thyroglobulin (Desialylated complex-type	Sigma	T1126 (Acid-
47	βGlcNAc	PAA	GlcNAcβ1-PAA	Glycotech	01-009
48	[6S]βGlcNAc	PAA	(6OSO$_3$)GlcNAcβ1-PAA	Glycotech	01-016
49	Agalacto-Fet	Glycoprotein	Agalacto fetuin (Agalactosylated complex-type N- and O-	Sigma	F3004
50	Agalacto-AGP	Glycoprotein	Agalacto α1-acid glycoprotein (Agalactosylated complex-	Sigma	G9885
51	Agalacto-TF	Glycoprotein	Agalacto transferrin (Agalactosylated complex-type N-	Sigma	T3309
52	OVM	Glycoprotein	Ovomucoid (Complex-type N-glycans)	Sigma	T2011
53	OVA	Glycoprotein	Ovoalbumin (Hybrid-type N-glycans)	Sigma	A2512
54	αMan	PAA	Manα1-PAA	Glycotech	01-005
55	βMan	PAA	Manβ1-PAA	Glycotech	01-050
56	[6P]Man	PAA	(6OPO$_4$)Manα1-PAA	Glycotech	01-006
57	INV	Glycoprotein	Yeast invertase (High mannose-type N-glycans)	Sigma	I4504
58	Tn	PAA	GalNAcα1-PAA	Glycotech	01-010
59	Core1	PAA	Galβ1-3GalNAcα1-PAA	Glycotech	08-023
60	Core2	PAA	Galβ1-3(GlcNAcβ1-6)GalNAcα1-PAA	Glycotech	01-083
61	Core3	PAA	GlcNAcβ1-3GalNAcα1-PAA	Glycotech	01-071
62	Core4	PAA	GlcNAcβ1-3(GlcNAcβ1-6)GalNAcα1-PAA	Glycotech	01-089
63	Forssman	PAA	GalNAcα1-3GalNAcβ1-PAA	Glycotech	01-026
64	Core6	PAA	GlcNAcβ1-6GalNAcα1-PAA	Glycotech	01-072
65	Core8	PAA	Galα1-3GalNAcα1-PAA	Glycotech	01-028
66	[3'S]Core1	PAA	(3OSO$_3$)Galβ1-3GalNAcα1-PAA	Glycotech	08-069
67	Galβ-Core3	PAA	Galβ1-4GlcNAcβ1-3GalNAcα1-PAA	Glycotech	01-116
68	Asialo-BSM	Glycoprotein	Asialo bovine submaxillary mucin (Tn)	Sigma	M3895 (Acid-
69	Asialo-GP	Glycoprotein	Asialo human glycophorin MN (T)	Sigma	A9791 (Acid-
70	STn	PAA	Neu5Acα2-6GalNAcα1-PAA	Glycotech	01-059
71	STn (Gc)	PAA	Neu5Gcα2-6GalNAcα1-PAA	Glycotech	01-107
72	ST	PAA	Neu5Acα2-3Galβ1-3GalNAcα1-PAA	Glycotech	01-088
73	Siaα2-6Core 1	PAA	Galβ1-3(Neu5Acα2-6)GalNAcα1-PAA	Glycotech	01-113
74	BSM	Glycoprotein	Bovine submaxillary mucin (Sialyl Tn)	Sigma	M3895
75	GP	Glycoprotein	Human glycophorin (Disialyl T and sialyl Tn)	Sigma	G5017
76	αGal	PAA	Galα1-PAA	Glycotech	01-003
77	Galα1-2Gal	PAA	Galα1-2Galβ1-PAA	Glycotech	01-056
78	Galα1-3Gal	PAA	Galα1-3Galβ1-PAA	Glycotech	01-018
79	Galα1-3Lac	PAA	Galα1-3Galβ1-4Glcβ1-PAA	Glycotech	01-075
80	Galα1-3LN	PAA	Galα1-3Galβ1-4GlcNAcβ1-PAA	Glycotech	01-079
81	Galα1-4LN	PAA	Galα1-4Galβ1-4GlcNAcβ1-PAA	Glycotech	01-110
82	Melibiose	PAA	Galα1-6Glcβ1-PAA	Glycotech	01-063
83	αGlc	PAA	Glcα1-PAA	Glycotech	01-001
84	βGlc	PAA	Glcβ1-PAA	Glycotech	01-002
85	Maltose	PAA	Glcα1-4Glcβ1-PAA	Glycotech	01-054
86	HA	BSA	Hyaluronic acid-BSA	Seikagaku	400720
87	CSA	BSA	Chondroitin Sulfate A-BSA	Seikagaku	400655
88	CSB	BSA	Chondroitin Sulfate B-BSA	Seikagaku	400660
89	HS	BSA	Heparan Sulfate-BSA	Seikagaku	400700
90	HP	BSA	Heparin-BSA	Calbiochem	375095
91	KS	BSA	Keratan Sulfate-BSA	Seikagaku	400760
92	αRha	PAA	Rhamnoseα1-PAA	Glycotech	01-008
93	Mannan (SC)	Glycoprotein	*S. cerevisiae* mannan	Sigma	M7504
94	Mannan (CA)	Glycoprotein	*C.albicans* mannan	Takara	MG001
95	Zymosan	Glycoprotein	Zymosan	Sigma	Z4250
96	Chitobiose	PAA	GlcNAcβ1-4GlcNAcβ1-PAA	Glycotech	08-057
97	BSA	BSA	-	Sigma	A7638
98	Negative PAA	PAA	-	Glycotech	01-000
